# Cryo-EM Structure of Mechanosensitive Channel YnaI Using SMA2000: Challenges and Opportunities

**DOI:** 10.3390/membranes11110849

**Published:** 2021-10-31

**Authors:** Claudio Catalano, Danya Ben-Hail, Weihua Qiu, Paul Blount, Amedee des Georges, Youzhong Guo

**Affiliations:** 1 Department of Medicinal Chemistry, Virginia Commonwealth University, Richmond, VA 23298-0540, USA; ccatalano@mymail.vcu.edu (C.C.); wqiu@vcu.edu (W.Q.); 2 Institute for Structural Biology, Drug Discovery and Development, Virginia Commonwealth University, Richmond, VA 23298-0113, USA; 3 Structural Biology Initiative, CUNY Advanced Science Research Center, New York, NY 10017, USA; danyabenhail@gmail.com; 4 Department of Physiology, University of Texas Southwestern Medical Center, Dallas, TX 75390-9040, USA; Paul.Blount@utsouthwestern.edu; 5 Department of Chemistry & Biochemistry, City College of New York, New York, NY 10017, USA; 6 Ph.D. Program in Biochemistry, The Graduate Center of the City University of New York, New York, NY 10031, USA

**Keywords:** YnaI, SMA2000, NCMN, cryo-EM, Mechanosensitive Channel

## Abstract

Mechanosensitive channels respond to mechanical forces exerted on the cell membrane and play vital roles in regulating the chemical equilibrium within cells and their environment. High-resolution structural information is required to understand the gating mechanisms of mechanosensitive channels. Protein-lipid interactions are essential for the structural and functional integrity of mechanosensitive channels, but detergents cannot maintain the crucial native lipid environment for purified mechanosensitive channels. Recently, detergent-free systems have emerged as alternatives for membrane protein structural biology. This report shows that while membrane-active polymer, SMA2000, could retain some native cell membrane lipids on the transmembrane domain of the mechanosensitive-like YnaI channel, the complete structure of the transmembrane domain of YnaI was not resolved. This reveals a significant limitation of SMA2000 or similar membrane-active copolymers. This limitation may come from the heterogeneity of the polymers and nonspecific interactions between the polymers and the relatively large hydrophobic pockets within the transmembrane domain of YnaI. However, this limitation offers development opportunities for detergent-free technology for challenging membrane proteins.

## 1. Introduction

Living organisms survive by relying on their adaptability to ever-changing environments. Many elegant mechanisms for adaptability have evolved in the long history of life on earth. Single-cell organisms such as bacteria often confront drastic osmolarity changes in their surroundings, leading to harmful turgor pressure changes on the bacterial cell membrane. Bacteria survival must keep the cell membrane turgor pressure in an acceptable range to avoid cell lysis [1,2,3]. It is well known that bacteria respond to this environmental change through mechanosensitive channels present on their cell membrane. However, activation mechanisms of mechanosensitive channels at the atomic level are fundamental questions that remain incompletely understood.

In bacteria, two major mechanosensitive families were identified and classified according to their conductance: the mechanosensitive channels of large conductance (MscL) and small conductance (MscS). In *Escherichia coli*, six different MscS-like channels were characterized: MscS (286 aa), MscK (1120 aa), YbdG (415 aa), YnaI (343 aa), YbiO (741 aa), and YjeP (1107 aa) [4,5,6]. The MscS-like channels are homoheptamers, with each monomer comprising a cytoplasmic domain and a transmembrane (TM) domain. The number of helices in the TM domain of the monomer varies across the family, viz., three (MscS), five (YbdG and YnaI), and eleven (YbiO, YjeP, and MscK). In addition, YbiO, YjeP, and MscK carry additions to their cytoplasmic domains [7,8].

Protein-lipid interactions play a crucial role in gating mechanosensitive channels [9,10,11,12,13,14]. Recently, Perozo and colleagues determined the cryo-EM structure of MscS in nanodisc in the near-native lipid bilayer environment. They discovered that “hook” lipids interact with the TM2-TM3 hairpin and play a role in force sensing. They also found that a bundle of acyl chains of lipid molecules occlude the permeation path [15]. Walz and colleagues systematically investigated the effects of different lipid species and used P-cyclodextrin to remove lipids from the nanodiscs to mimic the natural cell membrane tension on the MscS channel [16].

While substantial progress has been made in our understanding of MscS gating, we still understand little about the role and function of many other members of the MscS family. For instance, we do not know what gives these channels different gating thresholds and conductances. A potential reason is that the MscS family with larger transmembrane domains are more difficult to purify, reconstitute in vitro, and obtain structural data.

We reasoned that a limiting factor might have been the use of detergents to extract these proteins, which may have resulted in the irreversible denaturation of the proteins’ transmembrane region and possibly in the destabilization of the water-soluble domains as well [17,18]. Sometimes detergents can drive the protein far from its native-like structure, not only for the transmembrane region but also for the water-soluble region. More importantly, these effects are also seen in the juxtamembrane area [19]. For instance, several structures of YnaI have been solved by cryo-EM, using DDM, LMNG, and DIBMA as solubilizing agents [20,21,22]. However, these structures appear to be missing pore and hook lipids observed in MscS. In an attempt to circumvent those issues and shed light on the structure, function, and modulation of mechanosensitive channels of the MscS family, we have expressed, solubilized, and purified YnaI using the membrane-active polymer SMA2000 and solved their structure by cryo-EM.

We have successfully used the membrane-active polymer, SMA2000, to determine the high-resolution single-particle cryo-EM structure of a native lipid-bilayer patch with a membrane protein transporter, AcrB [23]. Our rationale was that by solubilizing these channels with SMA2000, their conformation and associated lipids would be retained. These attempts also allowed us to test whether this styrene-maleic acid copolymer could be of general use for the high-resolution structure determination of membrane proteins.

Here we report the cryo-EM structures of the SMA2000-purified wild-type YnaI at an overall resolution of 2.4 Å. We found seven native lipid molecules at the interface between the transmembrane and cytoplasmic regions of the structure. However, we were only able to partially resolve the structures of the transmembrane domain, which appeared very flexible and heterogeneous in the SMA2000 preparation. Together with the similar results obtained with MscS (to be reported separately), this points to limitations in using SMA2000 as a tool for solubilizing membrane proteins for biophysical studies.

## 2. Materials and Methods

### 2.1. Protein Expression and Purification for Cryo-EM

The wild-type low-conductance mechanosensitive channel-like (YnaI) was constructed into pRSFDuet-1 with an N-terminal deca-histidine tag. The plasmid was transformed into *Escherichia coli* strain BL21 (DE3) pLysS and grown on Luria broth agar plates in the presence of both ampicillin (100 mg/mL) and chloramphenicol (25 µg/L). The plates were incubated overnight at 37 °C. A single colony was chosen from each plate and transferred into 10 mL of Terrific broth (TB) in the presence of the antibiotics. The pre-culture was incubated at 37 °C and 220 rpm and kept shaking overnight. The following day, the cells were diluted 1:1000 in TB medium and grown at 37 °C to an OD600 of 0.8–1.0. The cell culture was cooled down to 20 °C, and protein expression was induced with 1.0 mM isopropyl β-D-1-thiogalactopyranoside (IPTG). The cells were grown overnight at 20 °C and then harvested by centrifugation at 7500× *g* for 15 min at 4 °C. Some cells were frozen at −80 °C for later use, and others were immediately resuspended in NCMN Buffer A and homogenized using a high-pressure homogenizer (Avestin EmulsiFlex-C3, ATA Scientific, Taren Point, Australia). The analyzed cells were then pelleted by centrifugation at 15,000× *g* for 30 min at 4 °C. Finally, the cell membrane was isolated by centrifugation at 250,000× *g* for 1 hour at 4 °C. Two grams of this membrane fraction were then resuspended in NCMN Buffer A and homogenized with a glass Dounce homogenizer. The membrane-active polymer SMA2000 (NCMNP1-1 as indexed in our NCMN polymer library) was mixed with the homogenized membrane fraction for a final concentration of 2.5% (*w*/*v*) and incubated for 2 h at 20 °C. Insoluble material was then pelleted down by ultracentrifugation (150,000× *g* for 1 h at 20 °C). The clarified supernatant was loaded onto a 5 mL Ni-NTA column (GE Healthcare, Chicago, IL, USA). After washing with 30 mL of NCMN Buffer B and 30 mL of NCMN Buffer C, the protein was eluted with 20 mL of a 1:1 *v*/*v* buffer mixture of NCMN Buffer C and NCMN Buffer D. Fractions containing YnaI were loaded onto a Superose 6 increase 10/300 column (GE Healthcare, Chicago, IL, USA) and eluted with 30 mL of NCMN Buffer E. YnaI fractions were pooled together and concentrated using Amicon Ultra 15 mL 30 kDa cut-off centrifugal filters (Millipore Sigma, Burlington, MA, USA) until the desired concentration. All washing and elution buffers contained 0.05% *w*/*v* of the polymer.

All buffers with compositions in detail are listed below:

NCMN Buffer A: 50 mM HEPES, pH 8.4, 500 mM NaCl, 5% glycerol, 20 mM imidazole, 0.1 mM TCEP

NCMN Buffer B: 25 mM HEPES, pH 7.8, 500 mM NaCl, 40 mM imidazole, 0.1 mM TCEP

NCMN Buffer C: 25 mM HEPES, pH 7.8, 500 mM NaCl, 75 mM imidazole, 0.1 mM TCEP

NCMN Buffer D: 25 mM HEPES, pH 7.8, 500 mM NaCl, 500 mM imidazole, 0.1 mM TCEP

NCMN Buffer E: 40 mM HEPES, pH 7.8, 100 mM NaCl, 0.1 mM TCEP

### 2.2. Negative-Stain Electron Microscopy

Briefly, 3.5 µL of the sample (~0.1 mg/mL) was applied to a glow-discharged 10 nm thick carbon grid; after 1 min, it was blotted with Grade 1 Whatman filter paper and briefly rinsed with a drop of 5 µL pure water. Rinses were repeated three times. The grid surface was then rinsed with two 5 µL drops of filtered 1% uranyl acetate consecutively, followed by another 5 µL drop of 1% uranyl acetate. After one min, the stain solution was then absorbed with a Whatman filter paper and dried in the air for another min. Negatively stained grids were stored in a grid box for later visualization on a 200 keV TEM (Tecnai F20, Thermo Fisher Scientific, Waltham, MA, USA) at 62,000× magnification at the specimen level.

### 2.3. Cryo-EM Specimen Preparation, Data Collection, and 3D EM Map Reconstruction

Samples were prepared for cryo-EM by applying 3 µL of freshly purified YnaI to glow-discharged holey carbon grids 1.2/1.3 with ultrathin carbon (300 mesh). The sample was blotted for 2 s with a force of 3 and then flash-frozen in liquid ethane and stored in liquid nitrogen using a Vitrobot Mark IV (Thermo Fisher Scientific, Waltham, USA) with the environmental chamber set at 100% humidity, 4 °C.

Cryo-EM specimen grids were imaged on a Titan Krios operated at 300 kV with a K2 Summit direct electron director (Gatan) in counting mode at 22,500× nominal magnification with a pixel size of 1.0733 Å/pix. The dose rate was 7.0 e^-^/Å^2^/s, with 40 frames s^−1^ collected for a total exposure time of 8.0 s and a final dose of 56 e^-^/Å^2^. An initial data set of 2155 micrographs for YnaI was obtained by automated data collection using Leginon, with nominal defocus values ranging between −1.5 and −2.5 µm [24]. Drift correction and dose weighting were performed using Motioncor2, and CTF correction was performed using CTFFIND4.1 [25,26]. A total of 330,000 particles were extracted, and several rounds of 2D classification were performed in Relion 3.0 [26,27]. The best-looking 2D classes and their respective particles were then subjected to 3D classification with no symmetry imposed. Three-dimensional auto-refinement was then performed on 142,000 particles with C7 symmetry imposed. The final map of YnaI had a resolution of 2.4 Å according to the gold-standard Fourier shell correlation. The local map resolution was calculated with ResMap [28].

### 2.4. Atomic Modeling, Refinement, and Validation

The map obtained with C7 symmetry was used to build and stereochemically refined atomic model for YnaI. An initial model was generated using the available EM structure of YnaI (PDB ID: 5Y4O) and placed into the sharpened density map using Chimera fit in the map [29]. The all-atom model of YnaI was built into the cryo-EM density using COOT [30]. Models were subjected to real-space refinement in Phenix with secondary structure restraints [31]. The additional densities were attributed to lipid molecules and built as phosphatidylethanolamine (PDB ID: PTY) in COOT.

### 2.5. YnaI Structural Comparison

Structures were downloaded from the Protein Data Bank (PDB), and Chimera was used for the structural comparison. The MatchMaker [32] extension of Chimera was used to superimpose the structures. The chains to match were explicitly specified, and the default settings were used: the Needleman–Wunsch algorithm with BLOSUM-62 and 30% weighting of the secondary structure with a gap penalty of 1. The sequence alignment was opened in Multialign Viewer, and the regions of interest were colored based on the RMSD values calculated for the alpha carbon atoms.

### 2.6. HINT Lipid-Protein Analysis

Hydrogens were added to the structures, and atomic charges were set with Gasteiger-Hückel, followed by proton-only energy-minimization using SYBYL-X-2.1.1 (Certara USA, Inc., Princeton, NJ, USA). The interatomic interactions between lipid and protein were evaluated using the HINT forcefield and score model [33,34] that uses atom-centered parameters, a_i_ and S_i_, the hydrophobic atom constant, and SASA, respectively, for atom i. Generally, a_i_ > 0 for a hydrophobic atom and a_i_ < 0 for a polar atom. S_i_ is larger for solvent-exposed frontier atoms but near zero for atoms at the center of functional groups. The score between atoms i and j is:

b_ij_ = a_i_ S_i_ a_j_ S_j_ T_ij_ e^−r^ + L_ij_,

where r is the distance (Å) between atoms i and j, T_ij_ is a descriptor function for polar-polar interactions, and L_ij_ is an implementation of the Lennard–Jones potential function. By convention, b_ij_ > 0 indicates favorable interactions, e.g., hydrophobic-hydrophobic or Lewis acid-Lewis base, and b_ij_ < 0 indicates unfavorable interactions, e.g., hydrophobic-polar or Lewis acid-Lewis acid.

## 3. Results

**Figure 1:**
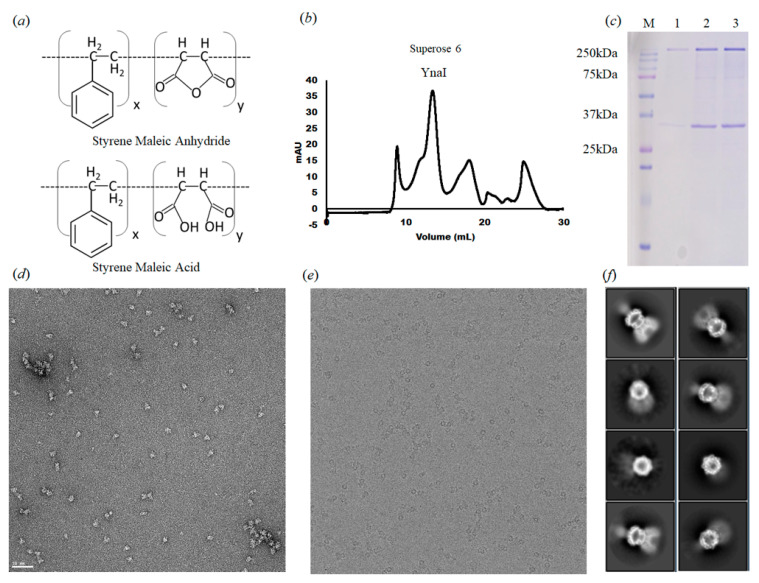
Purification and analysis of YnaI single particles. (**a**) SMA 2000 inactive form (styrene-maleic anhydride copolymer) and active form (styrene-maleic acid copolymer) (**b**) Size exclusion column purification profile of YnaI. (**c**) SDS-polyacrylamide gel electrophoresis (SDS-PAGE) analysis of YnaI. Lane M shows the protein marker (Bio-Rad prestained protein). YnaI is ~38 kDa (monomer) and ~265 kDa (heptamer). Lane 1–3: fractions of the second SEC peak. (**d**) Representative raw micrograph images showing negative-stain particles of YnaI. (**e**) Representative micrograph of YnaI on a cryo-EM thin carbon grid. (**f**) Representative 2D classes with side, top, and high angle views.

We overexpressed the full-length YnaI in *E. coli* strain BL21(DE3)pLysS and purified the protein with SMA2000 (Figure 1a) [23,35]. The gel filtration chromatography of YnaI did not show a symmetrical peak, probably due to its tendency to aggregate, as shown in the negative-stain micrograph (Figure 1b,d). The YnaI sample on the SDS-PAGE image shows both monomer and heptamer (Figure 1c). Two-dimensional classification of the cryo-EM data shows heptameric particles with well-defined cytoplasmic domains, but the transmembrane domain displays different conformations and order levels. Some of the side-view classes display a few well-defined transmembrane helices (Figure 1f). Table 1 summarizes the data collection and processing. All the refinement and validation statistics are available in Table 2.

### 3.1. The Structure of YnaI Solubilized with SMA2000 Shows Native Lipids Bound at the Juxtamembrane

**Figure 2:**
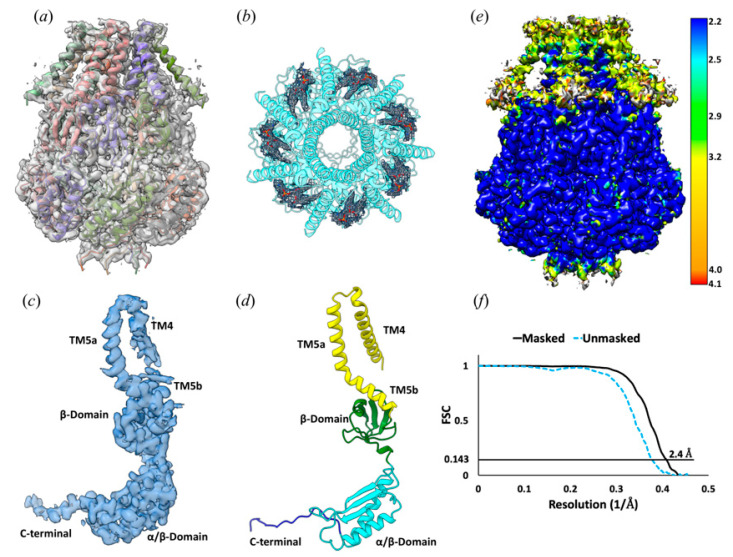
Cryo-EM structure of the YnaI channel. (**a**) the EM density at 2.4 Å resolution fitted with a YnaI heptamer, shown in cartoon representation. Each monomer is shown in a different color. (**b**) Seven PE molecules fitted into the density within the hydrophobic pockets between adjacent subunits of the YnaI heptamer. (**c**) The EM density at 2.4 Å resolution of one protomer. (**d**) Cartoon diagram showing the structural features of the YnaI monomer colored for each of the distinct domains. (**e**) The local resolution estimation of the YnaI map was calculated with ResMap. (**f**) FSC curve of the unmasked and masked map.

Consistent with the previously reported structures, 3D reconstruction of YnaI directly extracted from the cell membrane with SMA2000 reveals a heptamer. YnaI is paralogous to MscS with a similar architecture. YnaI’s most distinct feature is that its transmembrane domain contains five TMs instead of three for MscS. We could reconstruct the soluble portion for YnaI at high resolution; however, we could only resolve TM5 and TM4 of its transmembrane region (Figure 2). EM density for seven potential lipid molecules was found in the hydrophobic pockets defined by TMs 4–5 and the cytoplasmic region (Figure 2). In the *E. coli* inner cell membrane, phosphatidylethanolamine (PE) is the predominant lipid species, and this molecule also fits well into those densities [36]. This is also consistent with our lipid analysis results (section below). Therefore, we tentatively built PE molecules into the corresponding densities. Similar to our previous study on AcrB, we found no evidence of ordered SMA copolymer molecules around the transmembrane domain. Overall, YnaI appears in a closed state defined by the minimal diameter of the pore formed by TM5a ~13 Å, similar to the closed state of YnaI in DIBMA (14 Å) [37].

### 3.2. Protein-Lipid Interactions Affect Channel Conformation at the Juxtamembrane

**Figure 3:**
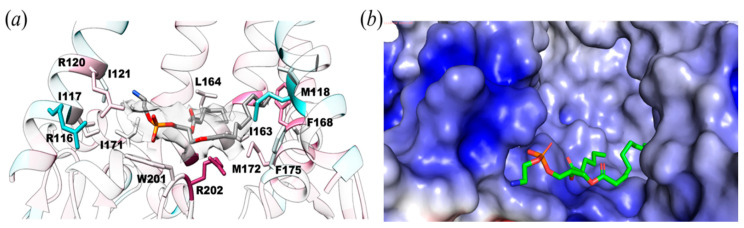
Protein-lipid interactions within 6 Å of YnaI. (**a**) Potential lipid molecule (density shown as transparent surface) interacts with R116, R120, I163, F167, M172, F175, W201, and R202. (**b**) Same orientation as in A but shown as the electrostatic surface of the binding pocket of lipid within YnaI that interacts with a lipid molecule. Electrostatic surface potentials were calculated using PyMol and APBS [38] with the non-linear Poisson–Boltzmann equation. Negatively and positively charged surface areas are colored in red and blue, respectively.

The lipid observed is bound in the juxtamembrane area defined by two TM5b helices and a loop from the β-domain, which forms a protein-lipid interface (Figure 3a). The TM5 kink (MscS TM3) marks the beginning of the second half of the TM5b helix, I163 to F175, which is amphipathic and lies parallel to the membrane bilayer. It functions as a conjunction between the membrane and the cytoplasmic domain. The electrostatic surface potential between the soluble and transmembrane regions shows protein-lipid interactions. The negatively charged phosphate head fits well within the positively charged pocket formed by R116, S119, R120, K123, Y174, W201, and R202 (Figure 3b).

**Figure 4:**
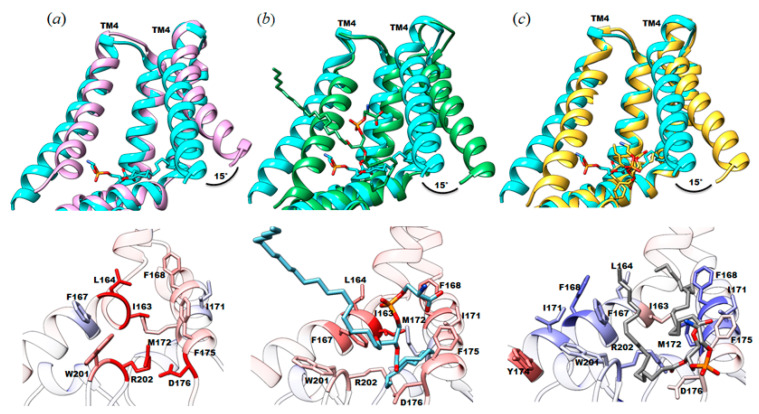
Structural comparison between the cryo-EM structure of SMA2000-YnaI with published structures focused on the protein-lipid interactions. Top, different orientation of TM4 between the SMA2000 structure (PDB ID: 7N4T, cyan) and (**a**) DDM structure reconstituted in amphipols (PDB ID: 5Y4O, purple); (**b**) LMNG structure (PDB ID: 6URT, green); and (**c**) DIBMA (PDB ID: 6ZYD, gold). Bottom, protein-lipid interactions comparison with specific amino acids colored by per-residue RMSD value. RMSDs less than 0.8 Å are shown in blue, RMSDs between 0.8 and 1.6 Å are shown in white, and RMSDs greater than 1.6 Å are shown in red.

Protein-lipid interactions were found within YnaI (Figure 4) and were quantified using the Hydropathic INTeraction (HINT) scoring tool that exploits LogP, a thermodynamic parameter representing solute transfer into a mixed solvent system (water and 1-octanol), to calculate the free energy of association. HINT analysis was conducted on the lipid-protein complex, in which all the non-covalent interactions were scored and ranked, giving a positive score for favorable and negative score for unfavorable interactions. Note that the HINT score has previously been shown to correlate well with binding free energy, i.e., ~500 HINT score units represent ΔΔG = −1 kcal mol^−1^ [37]. This analysis showed numerous favorable hydrophobic interactions between the tail of the lipid and M118, I121, I163, L164, F167, F168, I171, and M172 for a total of 1177 HINT units (~2.2 kcal mol^−1^). Likewise, the lipid head showed as many polar interactions, including hydrogen bonds, with W201, R202, and R120 for a total of 913 HINT units (~0.9 kcal mol^−1^). Considering both favorable and unfavorable interactions, the final score for our HINT analysis is 987, suggesting tight and strong interactions between the lipid molecule and the protein of about 2.0 kcal mol^−1^.

To quantitatively assess the differences in lipid stability between the different structures, we calculated the HINT score for the protein-lipid interactions in YnaI purified using DIBMA, a detergent-free method (PDB ID: 6ZYD), and YnaI purified with lauryl maltose neopentyl glycol (LMNG; PDB ID: 6URT), a detergent-based method. In the HINT analysis for the protein-lipid interaction of the LMNG structure, the O-{(R)-hydroxy[(2R)-3-(icosyloxy)-2-(tetradecanoyloxy) propoxy] phosphoryl}-L-serine (PDB ID: QGD) lipid molecule is scoring only 173 (~0.3 kcal mol^−1^) for favorable hydrophobic interactions (e.g., with F40, L96, F101, F175). This is probably due to the fact that one of the tails is sticking out into the solvent without making suitable interactions with any residue. In contrast, the head group of QGD scores 1269 (~2.5 kcal mol^−1^) for favorable polar interactions, of which the strong hydrogen bond with R120 (score = 620, ~1.2 kcal mol^−1^) particularly stands out. Even with this strong hydrogen bond, the overall score between the lipid and YnaI in LMNG is the lowest (209, ~0.4 kcal mol^−1^) among the three we analyzed due to its overall very unfavorable total hydrophobic/polar interactions. For the YnaI structure in DIBMA, the resolved PE has very strong favorable hydrophobic interactions (with V156, G160, I163, L164, F167, F168, M172, W201) (Figure 4c). Remarkably, due to the lipids’ geometries and placement, no hydrogen bonds were found between its head group and the protein, not even the R120 interaction that was found in both our reported structure and the LMNG one or the interaction with K108, since its sidechain is pointing away from the phospholipid head group (Figure 4).

Given that juxtamembrane lipid is bound to the same pocket in all three structures, we reasoned that the different orientations observed for those lipids might impact the conformation of this functionally important domain of the channel. To this end, we calculated root mean square deviation values between pairs of structures to highlight the areas of most change and made pairwise comparisons of those structures. RMSD values were calculated between our structure (PDB ID: 7N4T) and each of the above described structures (DDM (5Y4O) = 0.845 Å; LMNG (6URT) = 0.763 Å; DIBMA (6ZYD) = 0.622 Å). In all of them, the significant differences are located in the TM4 helix and the TM5b (MscS TM3b). In our structure of YnaI solubilized in SMA2000, TM4 (MscS TM2) is shifted by about ~15° or ~2.8 Å compared to the other published structures (Figure 4a–c). A distinct feature in the newly solved YnaI structure is the presence and position of the juxtamembrane lipid. We do not know whether this conformational difference is solely due to the different lipid positions.

## 4. Discussion

The gating of mechanosensitive channels depends on cell membrane tension [15,16,39,40,41]. However, the unique nature of these channels makes structural and functional study in vitro difficult and full of bias because of the difficulty of mimicking the natural lipid bilayer environment. The predominantly used detergent-based approaches damage the natural protein-lipid interactions that are crucial for understanding the conformational changes accompanied by opening and closing the channels [42,43]. Although nanodiscs have been used successfully for reconstituting MscS into a lipid bilayer environment, the initial purification of MscS still requires detergents, which could potentially remove essential native lipids from the transmembrane domain, i.e., hook lipids or pore lipids [15,42,43]. Further, the reconstituted lipid bilayer is artificial, which may be very different from the native structure of the cell membrane. The reconstituted lipids could also interact with the protein with different orientations and geometries, causing the stabilization of a non-native protein conformation. This is the case with AcrB, where we had reported a high-resolution single-particle cryo-EM structure of a lipid bilayer patch within the channel. Reconstitution of AcrB in nanodiscs could not faithfully restore this patch even using *E. coli* total lipids [44]. Thus, once protein-lipid interactions are damaged, they can be difficult to restore in vitro. On the cell membrane, membrane proteins have the advantage of having adequate time to interact with the native cell membrane lipid bilayer and thus form specific interactions at equilibrium. In vitro reconstitution is a very different process because the lipid environment is artificial; While we are accounting for some degree of membrane protein interactions with this approach, lipid nanodisc has significant limitations due to the absence of native lipids. Biological membranes are typically protein-crowded, and their lipid compositions vary in composition, i.e., PE, phosphatidylglycerol, cardiolipin, cholesterol, etc. Thus, it is probable that the local properties in the lipid bilayer significantly vary region by region, causing different interactions with the protein [45,46]. Therefore, biological membranes are more complicated than the lipid bilayer simulated within the lipid nanodisc. In principle, this process would not likely support the restoration of the natural lipid-protein interaction patterns.

### 4.1. Native Lipids Alter the Structure’s Morphology in the Membrane-Soluble Region Interface of YnaI

The structural analyses of the reported YnaI structures (Figure 4) revealed the importance of having a purification system able to co-purify native lipid molecules together with the protein. The presence of a lipid molecule in the structure can drastically change the model in the transmembrane region. Although the lipid is present in these three structures, the phospholipid head’s different orientation appears to provoke a drastic change in the TM4, causing a completely different orientation of this alpha-helix in our model. Therefore, lipid geometries and conformation are essential in stabilizing the final lipid-protein structure. The HINT scoring analysis supports this finding, which shows that the seven lipids in the structure reported here (PDB ID: 7N4T) contribute ~14 kcal mol^−1^ of the system stabilization, demonstrating their crucial role.

### 4.2. Limitation of SMA Copolymer

Although styrene-maleic acid copolymer SMA2000 enables us to determine cryo-EM structures of MscS and YnaI with some unique structural features, we also found a significant limitation of these polymers for membrane protein structural biology. We observed that SMA2000 could not maintain the transmembrane domains of mechanosensitive channels, MscS and YnaI, as stably as detergent or nanodiscs could [15,16,21]. These results point to this polymer’s potential limitations as a general “agent” resource for membrane protein research. SMA copolymers have well-known limitations, such as their incompatibility with divalent ions and low pH conditions [47]. Here, we point to another possible restriction of SMA copolymers: they appear to work well in maintaining the native structure and function of only a subset membrane proteins: proteins with more rigid transmembrane domains, such as AcrB, appear to be better suited to SMA copolymer solubilization than more flexible proteins, such as MscS and YnaI. Mechanosensitive channels have a flexible transmembrane domain and intricate protein-lipid interactions. We found that SMA copolymers (specifically SMA2000, this may also be true for other commercial SMA polymers) could extract YnaI single particles; however, they could not maintain the structure of the transmembrane domain in a stable conformation suitable for high-resolution structure determination. This could be explained by the heterogeneity of the SMA2000 polymer and nonspecific interactions between SMA2000 polymer molecules and the transmembrane helices. SMA molecules may be substituting for some lipid molecules in the lipid pockets and interacting with the transmembrane helices non-specifically. The SMA2000 polymer heterogeneity may yield very different interactions between SMA2000 and otherwise equivalent transmembrane helices. For YnaI, only the core TM helices TM4 and TM5 were resolved. Although some native lipids were retained in the transmembrane domain, many others may have been washed away similarly as from using detergents [21]. Compared with the DIBMA or LMNG solubilized YnaI, SMA copolymers fare worse in keeping the TM helices as ordered structures of this particular channel [21,22]. Many factors may cause the differences: the heterogeneity of SMA2000 polymers is much higher than the Anatrace grade of LMNG; The rigidity of packing of the polymer lipid-protein particles could be different between SMA2000 prepared sample and DIBMA prepared samples; the differences of the extraction efficiency between DIBMA and SMA2000 may lead to different polymer lipid-protein particle sizes and protein-lipid ratios.

### 4.3. The Need for a Large Membrane-Active Polymer Library for High-Resolution Structure Determination of Membrane Proteins

The necessity of structural information of native protein-lipid interaction in understanding the active mechanisms of mechanosensitive channels requires a detergent-free system for high-resolution structure determination. Detergents are not ideal for maintaining the native protein-lipid interactions [17,48]. While nanodisc reconstitution provided some protein-lipid interaction information, because of the artificiality of the reconstitution process, it is not guaranteed that native protein-lipid interaction can be restored precisely like that of the native cell membrane. A recent case study of AcrB demonstrated the limitation of nanodisc reconstitution compared with a detergent-free NCMN system [44]. However, although SMA2000 works well for AcrB, it did not work that well for mechanosensitive channels.

The failure to maintain the ordered structure of the transmembrane domain by SMA polymers may reflect the complexities of the cell membrane structure and the protein-lipid-SMA copolymer interactions. The complexity and variety of cell membrane systems from bacteria, fungus, plants, animals, and human cells make it extremely difficult, if not impossible, to obtain a single membrane-active polymer that can maintain the native structures of membrane proteins in their native cell environments. Each specific group of membrane proteins thus may need a particular class of membrane-active polymers for structural and functional studies due to this diversity. An extensive membrane-active polymer library will be required to identify the best membrane-active polymers for studying corresponding groups of membrane proteins.

### 4.4. Structural Study of Membrane Proteins Using SMA Copolymers Should Be Accompanied by Functional Validation

Functional studies using the SMA-extracted sample might not be relevant to the native structure. This may be especially true for membrane protein enzymes, channels, or transporters. Membrane protein activity may rely on the native structure and specific protein-lipid interactions. If the SMA copolymer damages the specific protein-lipid interactions and the native structures, the membrane protein may lose its function. For example, we found that KcsA can be purified with SMA2000. The particles appear to be homogenous; however, when SMA2000-prepared KcsA nanoparticles were reconstituted into proteoliposomes, we could not detect any channel activity using patch-clamp. On the contrary, when NCMNP7-1 polymer was used for proteoliposome reconstitution, the KcsA channel remained active [49]. This may reflect some limitations of SMA2000, and considering the incapability of SMA2000 in maintaining the ordered transmembrane domain of YnaI. It is possible that SMA2000 damaged the essential protein-lipid interaction within YnaI particles. Furthermore, while membrane proteins solubilized in detergent often precipitate, they rarely do so in the SMA polymer solution. This may be explained by the overall negative charges on the SMA polymers. However, while still kept in solution, the membrane proteins may not have retained their native and functional condition. The suitable solubility and stability may be deceptive and allow misplaced confidence in sample quality because we are used to evaluating the sample quality by the solubility and stability of membrane protein samples prepared using detergent-based approaches.

### 4.5. Mass Spectrometric Analysis of Lipids Using Samples from SMA Copolymer Prepared Samples Should Be Accompanied by Structural Validation

Ideally, membrane-active polymers can maintain membrane proteins in their native structures with concomitant native functions. If this is the case, mass spectrometry (MS) analysis of the protein samples may reveal native-associated lipid species on the membrane protein. However, this may not be true in practice because of the limitations of membrane-active polymers such as SMA. With the MscS or YnaI samples prepared with SMA copolymers, our structural analysis revealed that many lipids were not visible in the transmembrane domain. For example, the pore lipids located in the channel path of MscS appear crucial in keeping the mechanosensitive channel in a close state [15]. Without the pore lipid plug, the minimum diameter of the pore is about 9 Å, which is not compatible with a closed state. This might also be true for YnaI since the minimal pore diameter of YnaI is almost the same as that of MscS. If those lipids are indeed washed away, the lipid analysis of such samples will not reflect the true nature of the lipids associated with mechanosensitive channels in the native cell membrane, which is likely to be true for many other membrane proteins. To avoid biased interpretation of the protein-lipid interactions, we need high-resolution structures of studied membrane proteins to confirm the reliability of the sample for mass spectrometric analysis.

## 5. Conclusions

Currently, both detergent-based and detergent-free approaches such as DIBMA, SMALP, or NCMN system could not retain potential crucial lipid molecules, such as the hook lipids, pore lipids, onto the transmembrane domain of YnaI [20,21,22]. This presents a significant challenge for developing an understanding of its gating mechanism. However, this challenge is, at the same time, an opportunity. With the continuing rapid advancements of the SMALP, DIBMA, NCMN, and other detergent-free systems [44], hopefully, in the near future, we will be able to determine the single-particle cryo-EM structure of YnaI with all essential lipids intact.

## Figures and Tables

**Table 1:** Cryo-EM data collection and processing.

Microscope	Titan Krios (FEI)
Voltage (kV)	300
Detector	Gatan K2 SUMMIT
Nominal magnification	22,500
Electron exposure (e^-^ Å^-2^)	56.18
Defocus range (µm)	−0.1–3.1
Pixel size (Å^2^ per pixel)	1.07
Dose rate (e^-^/s/pixel)	7.02
Exposure time (s)	8
Movies stacks (no.)	2155
Boxsize (pixels)	256
Final particle images (no.)	142,000
Symmetry imposed	C7
Map resolution	2.4 Å
FSC threshold	0.143

**Table 2:** Structure refinement and validation statistics.

PDB ID	7N4T
EMDBID	EMD-24177
Non-hydrogen atoms	12,887
Protein residues	1603
Ligands	7
R.m.s. deviations	
Bonds (Å)	0.003
Angles (°)	0.483
Validation	
MolProbity score	1.72
Clashscore	5.48
Poor rotamers (%)	0.22
Ramachandran plot	
Most favored (%)	93.64
Allowed (%)	6.36

## Data Availability

The cryo-EM structure has been deposited in the Protein Data Bank with PDB ID: 7N4T. The EM map has been deposited in the Electron Microscopy Data Bank with EMDB ID: EMD-24177.
